# BUILDING SAFE ROUTES TO AGE IN PLACE THROUGH PARTNERSHIPS WITH AGE-FRIENDLY COMMUNITIES

**DOI:** 10.1093/geroni/igz038.947

**Published:** 2019-11-08

**Authors:** Holly Dabelko-Schoeny, Noelle L Fields, Ian Murphy, Katie White, Marisa Sheldon, Kristen E Ravi

**Affiliations:** 1 The Ohio State University, Columbus, Ohio, United States; 2 University of Texas at Arlington, Arlington, Texas, United States

## Abstract

Participation in active transportation by older persons has been associated with higher quality of life and longevity. Efforts across the United States to increase active transportation (walking, biking and fixed route transportation) by older adults have lacked meaningful input by older adults, are non-theoretical and have had inconclusive results. This paper describes the findings from Phase 1 of the Safe Routes to Age in Place project, a collaboration between Age-Friendly Franklin County, The Ohio State University (OSU) and the University of Texas-Arlington (UT-A), funded by the Ohio Department of Transportation. The aim of the study is to increase the options and safe utilization of active transportation by older persons in three pilot neighborhoods in Franklin County, Ohio utilizing community-based participatory action research strategies with a customized data collection app, MyAmble. The purpose of Phase 1 was to engage city and regional planning students, government officials and older adults to identify “hot spots” in the three pilot neighborhoods. Heat mapping of community data including locations of densely-populated vulnerable older adults, fixed transportation routes, bus stop signage and benches, shared bike stations, bike routes, paratransit routes and pedestrian crash data was completed. Maps were used to identify areas of focus and discussions with municipal leaders and older adult residents further refined target areas to be used in the second phase of the study. Older residents completed walk audits using the MyAmble app and identified issues with sidewalks, crosswalk timers and bus stops. Implications related to age-friendly communities and active transportation are offered.

